# The Clinical Potential of Circulating Tumor Cells; The Need to Incorporate a Modern “Immunological Cocktail” in the Assay

**DOI:** 10.3390/cancers5041739

**Published:** 2013-12-13

**Authors:** Jonathan W. Uhr

**Affiliations:** Cancer Immunobiology Center, University Texas Southwestern Medical Center, Dallas, TX 75390, USA; E-Mail: jonathan.uhr@utsouthwestern.edu

**Keywords:** CTCs, picomolar affinity, scFv, EpCAM, early diagnosis

## Abstract

The accepted clinical assay, CellSearch^®^, and lab-on-a-chip tests for capturing circulating tumor cells are antibody-mediated. Attempts to improve their sensitivity have relied upon physical changes in the instruments. There have been no significant advances in improving the antibody-mediated portion of the capture. Modern immunologic engineering offers major possibilities for improving the sensitivity and other features of the assay. These include obtaining univalent antibody fragments such as scFvs with picomolar binding affinity and sufficient specificity; altering them to enhance their range of potential contact with target antigens; using antibodies directed against different epitopes on epithelial, mesenchymal or organ-specific cell surface markers to allow simultaneous binding and investigating non-antibody binding molecules as substitutes for antibody. These maneuvers could markedly improve the ability of current assays to improve patient care and might result in an acceptable test for detecting cancer earlier in high risk patients.

## 1. Introduction

The identification of circulating tumor cells (CTCs) marked the beginning of an intensive analysis of these cells for clinical purposes [1]. The initial studies were based on immunophenotype, cytomorphology and aneuploidy. In breast cancer patients, CTCs always had a similar clone in the primary tumor as judged by the pattern of aneuploidy [2]. CTCs were detected in approximately 50% of patients with T1, N0,1, M0 breast cancers [1,3]. Approximately 40% of patients who were clinically disease-free 7–22 years after mastectomy had low levels of persisting CTCs [4]. Because of their rapid turnover in blood, this unexpected finding indicates that there are mechanisms that balance replication and death of disseminated cancer cells, thereby, controlling the cancer in the absence of further treatment. Whether these mechanisms are related to the immune system [5], angiogenesis [6] or to processes controlling organ size [7] will be important in designing new targeted and less toxic therapeutic regimens to convert breast and perhaps other cancers to chronic diseases that persist without relapse for decades.

These early studies were used by Veridex Inc. (Veridex, Raritan, NJ, USA) to develop CellSearch^®^, a semi-automated imunomagnetic instrument that is the only method for counting and characterizing CTCs currently approved by the FDA. The number of CTCs detected after a recurrence was shown to correlate with disease severity and prognosis in several recurrent carcinomas and helped predict the efficacy of further treatment [8,9,10]. A plethora of clinical studies using this instrument are currently in progress to further improve prognostication and treatment decisions [10,11]. In addition to more extensive immunophenotyping, in depth genetic analyses of individual CTCs [12] is providing important new insights concerning progression of cancer as well as potential therapies. However, CellSearch^®^ lacks the sensitivity of its prototype [1] and only detects a sufficient number of CTCs for clinical purposes in 50%–70% of patients with recurrent carcinomas [13]. It is essential to develop a far more sensitive capture method.

A variety of new technologies for CTC-capture have been developed, in particular, microfluidics approaches [14] such as lab-on-a-chip [15]. These antibody-coated chips facilitate interactions between CTCs and the surface of the chip using micro devices with induced vortex-flow. The early results are promising but the instrumentation is not yet at the stage to replace CellSearch^®^, and, it is not sufficiently sensitive to routinely detect early-stage carcinomas. 

## 2. Specific Aim

The purpose of this article is to alert investigators in the CTC-field regarding the potential of modern immunologic engineering for increasing the sensitivity of capture whether using metal particles or a microfluidic chamber. These include use of single chain, univalent, scFv antibodies (Fv) with picomolar affinity anchored to a metal particle by a short flexible polypeptide chain. Ideally, multiple Fvs against different cell surface molecules and/or multiple epitopes on the same molecule would result in an immunological cocktail specific for each cancer-type or for general diagnostic purposes. Such a cocktail should increase the sensitivity of antibody-mediated assays for detecting CTCs and lead to improved methods for early diagnosis, prognostication; and evaluation of effectiveness of therapeutic regimens.

## 3. Discussion

### 3.1. Production of Picomolar-Affinity Antibodies

It would seem self-evident that an antibody with a very high on- and a very low affinity off-rate should increase sensitivity of an antibody-mediated method to capture CTCs. The binding affinity of antibodies produced in a secondary response in experimental animals is frequently measured from 10^7–9^ K_d_. It is now possible to achieve binding affinities that are several orders of magnitude more sensitive. This is readily accomplished by producing a phage antibody library using *in vitro* stimulation and selective methods using B cells from non-immunized animals (see Appendix A1). The result is the production of antibodies with picomolar affinities [16,17,18,19].

### 3.2. Specificity of High Affinity (HA) Antibodies

An immediate concern with their use is their specificity. This concern is heightened because secondary antibody responses in animals that can result in higher affinity antibodies typically increase cross-reactivity with homologous, related antigens. It is critical to eliminate capture of non-CTCs in the assay. However, some of the HA antibodies described above have increased specificity. For example, HA-anti-botulinum neurotoxins directed against each of the 4 different serotypes did not cross-react with the others despite 32%–59% sequence homology [20]. An Fv specific for *C. trachomatis* stains its corresponding elementary bodies in infected cells while neighboring uninfected cells remain unstained [20]. It is irrelevant whether these examples are common or rare. For CTC capture, it is only necessary to select from HA-antibodies those that do not cross-react with unwanted antigens. 

It is also possible to increase specificity of antibodies after their selection using structure-based computational design as has been done to increase affinity [21] (see Appendix A2). This approach was used to construct synthetic molecules that were 2% as large as the original antibodies and which had nM and pM affinities. They bound only to cell lines expressing the specific antigen [22]. Using saturated mutagenesis, the specificity of an antibody against progesterone with pM affinity was improved 23-fold over the original antibody in discriminating against 5 β-dihydroprogesterone without loss of its pM affinity [23]. In summary, there is no evidence to exclude obtaining HA antibodies with acceptable specificity for CTC-capture.

### 3.3. Fv Antibodies

Fv is a univalent antibody consisting of the V_H_ and V_L_ antigen binding sites held together by a polypeptide chain. It is particularly attractive for capture of CTCs because of its small size, and partial flexibility. Using the EpCAM molecule, a favored target for capturing CTCs, as an example; it has a molecular weight of 35,000 [24,25]; its crystal structure has not been determined. Hence, it is possible to visualize several Fv antibodies specific for different subregions of EpCAM binding simultaneously. However, there are many issues regarding their availability. The *C*-terminal portion of the molecule extends into the cytoplasm and is hidden. There are typically three glycosylation sites on EpCAM. The extent of oligomerization of EpCAM as it reaches the cell surface and its effect on availability of subregions to antibody are not known. However, prior studies with divalent antibodies [26] indicate that antibodies can be produced against both the *N*-terminal and a 2nd subregion nearer to the cell surface. 

With regard to an immunomagnetic assay, the access of Fv antibodies against all the theoretically possible subregions of EpCAM would be sterically inhibited by the large size of the metal particle compared to the sizes of the Fv and the EpCAM-subregions. The questions arises as to how such access can be facilitated? The super magnetic particle can be made smaller if the distance between it and the magnet is shortened, and/or the magnetic field is made stronger. A short flexible peptide can be attached to the metal particle and to the peptide linking the H and L chains of the Fv. The array of antibodies against different subregions of EpCAM would have to be dense so that there are Fvs of appropriate specificity within reach of their epitopes. The result would be a magnetic particle with short flexible antibody tentacles, an “immunological octopus”. This should result in an increase in sensitivity because of an increased rate of collisions caused by increased participants and increased flexibility plus a major increase in successful collisions because of the extreme increase in avidity resulting from several more picomolar affinity Fv-EpCAM interactions [27].

### 3.4. Other Potential Target Molecules on CTCs

For breast cancer, there are additional cell surface target molecules: Mucin-1, EGFR, c-MET. Trop-2, *N*-Cadherin, CD 138, CD 318, protein receptor, MSC, Twist, mammaglobin, HER-1,2,3 and 4, *etc*. The difference in the concentration of CTCs among cancers from different organs and the heterogeneity of CTC-markers makes it difficult to determine how many biomarkers will be optimal for general or organ-specific tumor detection. For example, the recent description of Plastin 3 as a hitherto unknown but important epithelial-mesenchymal transition (EMT) marker in colorectal cancer is illustrative [28]. Possibly, several specificities to EpCAM, to EMT antigens [29] and to organ specific ones for high risk patients might be optimal if the primary need is for HA antibodies and for multi-hits to target antigens. 

### 3.5. Non Antibody Molecules for Specific Binding

Potential substitutes for Fv antibodies in the above assays are novel non-antibody scaffolds for selective binding. One approach consists of a modular recognition of peptides, amino acid by amino acid, from preselected modules [30,31]. These peptides have sequence-specific binding. Libraries of repeat proteins are produced. Then, using a combination of design, directed evolution and structure determinations by crystallography and NMR, appropriate engineering can result in a protein that binds specifically to a target antigen with high affinity [32]. They are stable; can be prepared in very large amounts from *E. coli* and show affinities in the picomolar range. One group has focused on Designed Ankyrin Repeat Proteins (DARPins) [33,34]. They selected DARPins from combinatorial libraries by phage display. Using random mutagenesis and ribosome display selection, binders with picomolar affinity (e.g., K_d_ = 68 pM) were obtained.

Similar results have been reported using RNA and DNA scaffolds termed “Aptamers” [35,36]. These are single-stranded molecules of about 40 nucleotides in length with unique conformations based on hairpin formation and additional three-dimensional interactions resulting in specific target recognition. Their small size is attractive. An anti-EpCAM RNA aptamer of only 19 nucleotides has been described [37]. One potential problem is their high negative charge which may restrict their ability to reach particular epitopes on cell surface target molecules. 

HA binders can also be obtained by combining features of Ig and non-Ig binding domains. Thus, a combination of a llama Ig heavy chain fused to a 57 amino acid SH_3_ domain bound to its target, the HIV-1 Nef protein, with sub-picomolar affinity (K_d_ 0.54 pM) [38]. These are exciting new fields but it is not yet clear whether the scaffolds will be more effective than engineered Fv in capturing CTCs.

## 4. Conclusions

Immunological engineering has revolutionized the capacity to obtain small univalent antibody fragments that bind to linear or conformational epitopes with extremely high-on and extremely low-off rates. Such antibodies against epithelial, EMT and organ specific cell surface molecules should be immediately evaluated in CTC immunoassays. Alternate ways of attaching such antibodies to solid surfaces and different molecular types for specific binding should also be investigated. There is every reason to hypothesize that these maneuvers alone should increase the sensitivity of the assays and facilitate the ability to detect early cancers in high risk groups and eventually in the whole population. 

I have stressed that a more sensitive assay must be obtained to reach such a goal. The appropriate perspective for clinical scientists is to emphasize that there are many issues to be resolved, particularly, the need to have virtually no false positives. This is particularly pertinent since colonic inflammation, and, possibly, inflammation from other organ sites, can cause normal epithelial cells to circulate in some patients [39,40]. Also, the clinical significance of finding CTCs in previously undiagnosed individuals of different ages is not known. As mentioned above, CTCs can be observed in late survivors of mastectomy at low risk of recurrence. These considerations indicate the need for continued interrogation of CTCs in the future when more sensitive assays change the numbers and, possibly, the types of CTCs captured or when testing a new population of previously undiagnosed individuals [41]. However, it is also possible that a more sensitive assay will detect unexpected numbers of CTCs above a cutoff point for false positives in a significant portion of patients with early cancer. Thus, there should be no delay in appropriate clinical trials when a sufficiently improved assay is finally developed.

## Appendix

### A1

Antibodies can be synthesized entirely *in vitro* or can be produced by activated B lymphocytes from non-immunized animals. For the latter purpose, a very large number of different antibodies are made by stimulation with immunogens of interest with directed efforts to increase binding affinity. The result is an antibody library containing antibodies with picomolar affinity to the antigen. The formation of a typical library is as follows: Total RNA is prepared from animal lymphoid cells; cDNA is then synthesized from total RNA. Recombinant antibodies are constructed from the variable (V) region genes of both L and H chains; Fab fragments or single chain scFv (Fv) antibodies that consist of the antigen-binding regions and connected by a flexible peptide linker are constructed and encoded by a single gene. A phage antibody library is created by cloning these genes as fusion proteins with a minor coat protein of bacteriaphage. Each resulting phage has a functional antibody protein on its surface and contains the gene encoding the antibody incorporated into the phage genome. Particular phage antibodies that specifically bind to a protein of interest e.g., EpCAM can be separated from nonbinding phage antibodies with affinity chromatography techniques. The antibody genes are cloned and the antibody fragments are expressed in *E. coli*, The number and affinity of the antibodies generated against a particular antigen is a function of library size and diversity with larger libraries yielding a greater number of high affinity (HA) antibodies. Libraries containing as many as 10^11^ antibodies have been obtained. Antibodies from the phage library can then be affinity selected by the use of decreasing concentrations of soluble antigen for stimulation and/or by mutagenesis affecting either the hypervariable regions which act as contact residues for the antigen, or by inducing subtle changes in the framework. HA antibodies up to 1 pM can be produced.

### A2

Use of structure-based computational methods to optimize the binding affinity or specificity of an antibody fragment or a non-antibody binder. The crystal structure of the antibody interacting with the antigen or a scaffold-antigen interaction is determined. The contacts are noted. The rest is mathematical. Mutagenesis “experiments” are performed to seek higher affinity- or higher selectivity-mutations on the contact residues or other possible residues that could affect the specific binding. At this point, computational protein design is used which rests on techniques to sample a large number of new designs and the ability to accurately predict the properties of the designs. A very large number of algorithms have been developed involving amino acid types and side chains that allow the conformations at many positions to be screened for the best design. Energy of the interaction is evaluated. It is possible to find the best designs in a virtual library of approximately 10^40^ possibilities within several days. This is a powerful technique for further refinement of a specific binder.
